# Hesperidin Attenuates Lead‐Induced Cognitive Impairment via TFEB Activation: Implications for Neuroprotection in Alzheimer’s Disease

**DOI:** 10.1002/alz70859_104758

**Published:** 2025-12-26

**Authors:** Varinder Singh, Pratham Gautam

**Affiliations:** ^1^ Department of Pharmaceutical Sciences and Technology, Maharaja Ranjit Singh Punjab Technical University, Bathinda, Punjab India; ^2^ Maharaja Ranjit Singh Punjab Technical University, Bathinda, Punjab India

## Abstract

**Background:**

Lead exposure is a major environmental neurotoxin that contributes to cognitive impairment through oxidative stress, inflammation, and mitochondrial dysfunction. This study investigates the role of transcription factor EB (TFEB) in the neuroprotective effects of hesperidin against lead‐induced cognitive dysfunction.

**Method:**

Wistar rats of either sex were administered lead acetate (100 mg/kg, p.o.) once daily for 30 days to induce memory deficits. Hesperidin (40 and 80 mg/kg, p.o.) was administered 30 minutes post‐lead exposure. Cognitive function was assessed using the Morris Water Maze and Novel Object Recognition tests. Biochemical analyses included oxidative stress markers (reduced glutathione, TBARS), inflammatory mediators (TNF‐α, IL‐6, NF‐κB), and mitochondrial function (complex I and II activities). Histopathological evaluation using H&E staining was conducted to assess neuronal architecture in the hippocampus.

**Result:**

Hesperidin treatment significantly improved memory performance in behavioral tests, restored GSH levels, enhanced mitochondrial complex I and II activities, and reduced TBARS, TNF‐α, IL‐6, and NF‐κB levels. Histopathological analysis revealed increased neuronal cell count and reduced neuronal pyknosis and vacuolation in hesperidin‐treated groups. However, these protective effects were abolished upon co‐administration of the TFEB inhibitor (eltrombopag), indicating the essential role of TFEB activation in hesperidin‐mediated neuroprotection.

**Conclusion:**

These findings suggest that hesperidin alleviates lead‐induced cognitive dysfunction by mitigating oxidative stress, inflammation, and neuronal damage while enhancing mitochondrial function, potentially through TFEB activation. This study highlights the therapeutic potential of hesperidin in heavy metal‐induced neurotoxicity and cognitive disorders.

